# P-983. Online CME Improves Knowledge, Competence and Confidence in the Management of Hepatitis B among Primary Care Physicians

**DOI:** 10.1093/ofid/ofae631.1173

**Published:** 2025-01-29

**Authors:** Sara Thorpe, Lindsey Gardner, Maria B Uravich

**Affiliations:** Medscape Education, New York, New York; WebMD Health Corp, Newark, New Jersey; Medscape Education, New York, New York

## Abstract

**Background:**

Viral hepatitis, affecting diverse populations, is a significant public health concern with 850,000 patients living with hepatitis B virus (HBV). Public policy initiatives, such as the CDC's Division of Viral Hepatitis 2025 Strategic Plan, aim to globally eliminate viral hepatitis through culturally relevant prevention, testing, and treatment services. Healthcare providers need professional education to increase awareness, enhance capacity for reducing infection rates, improve identification and linkage to treatment, and address healthcare disparities. This study examined whether online continuing medical education (CME) could improve the knowledge and competence of primary care physicians in hepatitis B virus (HBV) screening, testing, and vaccination into clinical management.

**Methods:**

The CME intervention comprised of a 30- minute online video-based, three-faculty panel discussion. Response to 3 multiple choice, knowledge questions 1 self-efficacy, 5-point Likert scale confidence question were analyzed using a repeated pairs pre-/post-assessment study design. Pre- to post responses were compared using a McNemar’s test to assess statistical significance (P < .001 level). The activity posted on 6/12/2023; data were collected through 8/24/2023.

**Results:**

The analysis set consisted of responses of primary care physicians (n=193). Analysis demonstrated a significant improvement in knowledge, competence, and confidence.

• 65% relative increase in knowledge regarding ACIP Vaccine guidelines (34% pre vs. 56% post, P< .001)

• 18% relative increase in knowledge of CDC Hepatitis B guidelines (66% pre vs. 78% post, P< .001)

• 17% relative increase in competence related to overcoming barriers to Hepatitis B vaccination (65% pre vs. 76% post, P< .001)

• 35% improved confidence related to recommending HBV screening in primary care settings (P< .001)

**Conclusion:**

This study demonstrated the success of online, video-based round table discussion CME on improving knowledge, competence and confidence related to HBV management in primary care. Education enhanced primary care physicians integration of updated Centers for Disease Control and Prevention (CDC) guidelines for hepatitis B virus (HBV) screening, testing, and vaccination into clinical management.

**Disclosures:**

**All Authors**: No reported disclosures

